# Risk Prediction Models for Early ICU Admission in Patients With Autoimmune Encephalitis: Integrating Scale-Based Assessments of the Disease Severity

**DOI:** 10.3389/fimmu.2022.916111

**Published:** 2022-06-10

**Authors:** Chunmei Wu, Yongkang Fang, Yingying Zhou, Huiting Wu, Shanshan Huang, Suiqiang Zhu

**Affiliations:** Department of Neurology, Tongji Hospital, Tongji Medical College, Huazhong University of Science and Technology, Wuhan, China

**Keywords:** autoimmune encephalitis, intensive care unit, risk factor, prediction, model

## Abstract

**Background:**

In patients with autoimmune encephalitis (AE), the prediction of progression to a critically ill status is challenging but essential. However, there is currently no standard prediction model that comprehensively integrates the disease severity and other clinical features. The clinical assessment scale in autoimmune encephalitis (CASE) and the modified Rankin Scale (mRS) have both been applied for evaluating the severity of AE. Here, by combining the two scales and other clinical characteristics, we aimed to investigate risk factors and construct prediction models for early critical care needs of AE patients.

**Methods:**

Definite and probable AE patients who were admitted to the neurology department of Tongji Hospital between 2013 and 2021 were consecutively enrolled. The CASE and mRS scores were used to evaluate the overall symptom severity at the time of hospital admission. Using logistic regression analysis, we analyzed the association between the total scores of the two scales and critical illness individually and then we evaluated this association in combination with other clinical features to predict early intensive care unit (ICU) admission. Finally, we constructed four prediction models and compared their performances.

**Results:**

Of 234 patients enrolled, forty developed critical illness and were early admitted to the ICU (within 14 days of hospitalization). Four prediction models were generated; the models were named CASE, CASE-plus (CASE + prodromal symptoms + elevated fasting blood glucose + elevated cerebrospinal fluid (CSF) white blood cell (WBC) count), mRS and mRS-plus (mRS + prodromal symptoms + abnormal EEG results + elevated fasting blood glucose + elevated CSF WBC count) and had areas under the ROC curve of 0.850, 0.897, 0.695 and 0.833, respectively. All four models had good calibrations. In general, the models containing “CASE” performed better than those including “mRS”, and the CASE-plus model demonstrated the best performance.

**Conclusion:**

Overall, the symptom severity at hospital admission, as defined by CASE or mRS, could predict early ICU admission, especially when assessed by CASE. Adding other clinical findings, such as prodromal symptoms, an increased fasting blood glucose level and an increased CSF WBC count, could improve the predictive efficacy.

## Introduction

The severity of autoimmune encephalitis (AE) is highly heterogeneous because it can range from mild impairments in working memory to the most severe, persistent disorders of consciousness that would require lasting care or could even cause death (1–3). During the acute stage of the disease, the rapid progression of an immune inflammatory response may cause severe neurological deficits, status epilepticus, coma, and respiratory failure (4). Moreover, with a high risk of suffering from multiple concurrent complications, such as lung infections and sepsis, the reported mortality of AE was as high as 40% in some studies (1, 4). Therefore, some patients require admission to intensive care units (ICUs) for the maximum standard of care. It is still unknown why some patients with AE survive the acute phase of the disease, while others are overwhelmed by the life-threatening acute phase. Previously, several variables, such as anemia, a definite diagnosis of AE (5), cerebrospinal fluid (CSF) WBC >20 cells/mm3 (6), failure of first-line immunotherapy (7) and a high CSF IL-17A concentration (8), were found to be associated with critical illness and subsequent ICU admission. However, to date, there is no standard prediction model that comprehensively includes both the clinical symptom severity and laboratory tests. For the subset of the AE patients who are critically ill, delayed admission to the ICU may be an independent risk factor for poor outcome (9). Therefore, identifying the risk factors for the deterioration to critical illness is crucial for early administration to the intensive care and for timely therapeutic implementation in order to improve prognosis.

Scales are ubiquitously used to assess the severity of symptoms in neurological diseases (10–12). Due to the lack of customized scales, the modified Rankin Scale (mRS) is usually applied for evaluating the neurological severity and outcomes in AE patients (2, 13, 14). However, mRS was originally designed to measure disability after stroke, and it was weighed toward motor deficits and functional independence and apparently with shortage in measuring the non-motor symptoms that frequently occur in AE (15). The clinical assessment scale in autoimmune encephalitis (CASE) is a novel tool that was developed in 2019 to specifically evaluate the clinical severity of a series of syndromes, including definite AE, definite autoimmune limbic encephalitis (ALE), autoantibody negative but probable AE, definite acute disseminated encephalomyelitis (ADEM), and definite and probable brainstem encephalitis (16). The CASE is composed of nine major clinical features of AE, with a total score ranging from 0 to 27 (16), and this makes it a fine quantification tool that has great potential in the assessment of AE. Two studies that evaluated Chinese patients with antibody-positive AE confirmed the accuracy of the clinical evaluation of CASE (17, 18). However, CASE has not been popularized in clinical practice, nor is there a study comparing the performance of the mRS and CASE in predicting ICU admission independently or in combination with other clinical factors.

Prolonged hospital length of stay may increase the risk of hospital-acquired infection (19), leading to an increased likelihood of ICU admission for reasons not directly related to AE. Therefore, to be representative of ICU admission for AE-related reasons, this study aimed to investigate the association between symptom severity at hospital admission, as assessed by the CASE or mRS score, and early deterioration, requiring ICU care, in patients diagnosed with definite and probable AE, and to construct scale-based risk prediction models and compare the performances of these models. Finally, we explored whether the addition of other clinical factors to the models that evaluated symptom severity could improve their predictive efficacy.

## Materials and Methods

### Patients

We retrospectively extracted data from the medical records of consecutive patients diagnosed with encephalitis who were treated from January 2013 to October 2021 in the Department of Neurology, Tongji Hospital, and we screened those patients who met the clinical diagnostic criteria for definite and probable AE proposed by Mittal and Graus et al. in 2016 (3). Specifically, the patients with definite AE, definite ALE and autoantibody-negative but probable AE were included in this study. The detailed diagnostic criteria for each AE are described in the **Supplementary Table 1**. Patients were excluded when 1) they had infectious encephalitis with laboratory evidence, including tuberculosis or bacterial, fungal, viral (IgM), or parasitic infections; format correction: 2) they did not fulfill the probable AE criteria (e.g, AE mimics such as Creutzfeldt–Jakob disease, metabolic encephalopathy, neoplastic disorders and cerebrovascular disease (Identification of these disorders was based on history, physical examination, laboratory tests, and auxiliary tests; auxiliary tests used are listed in **Supplementary Table 2**); other diseases screened from the electronic database such as meningitis); 3) they had received immunotherapy before hospital admission or this admission was not their index AE admission; 4) they were under the age of 18; 5) they were admitted to the ICU immediately at hospitalization; or 6) they had incomplete medical records. Early ICU admission was defined as admission to the ICU at any time point within two weeks of hospitalization and patients admitted to the ICU beyond 14 days of hospitalization were classified into “non-early ICU admission” group. The protocol was approved by the institutional ethics board of Tongji Hospital, Tongji Medical College of Huazhong University of Science and Technology (ID: TJ-IRB20211221).

### Data Collection

The following details about the acute phase of the disease were obtained by 3 neurologists (W-CM, W-HT and Z-YY): (1) demographic information (sex, age); (2) clinical features: comorbidities including hypertension, diabetes and autoimmune diseases; prodromal symptoms such as fever, headache, nonspecific respiratory or gastrointestinal symptoms and other nonspecific viral-like symptoms; the symptoms at onset and all of the symptoms that were present from the onset to hospital admission; the date of onset, the date of hospital admission and discharge; (3) laboratory results: the results of blood tests within 24 hours and the first laboratory CSF sample analysis after admission. For antibody detection, blood and CSF samples were sent to the same laboratory for detection of antibody types and titers using cell-based assay (CBA) in an indirect immunofluorescence (IIF) test and immunospot assay. Antibody titer was defined as low (+, 1:10 in blood or 1:1 in CSF), moderate (++, ≤1:100 in blood or ≤1:10 in CSF), or high (+++, ≥1:320 in blood or ≥1:32 in CSF), with initial dilution titers of CSF and serum of 1:1 vs. 1:10. Six basic types of antibodies were detected for every patient: anti-N-methyl-D-aspartate receptor (NMDAR) antibody, anti-α-amino-3-hydroxy-5-methyl-4-isoxazolepropionic acid 1 (AMPA1) receptor antibody, and anti-AMPA2 receptor antibody, anti-leucine-rich glioma-inactivated 1 (LGI1) antibody, anti-gamma-aminobutyric acid-B receptor (GABA_B_R) antibody and anti-contactin-associated protein-like 2 (CASPR2) antibody. Other optional antibody types including anti-GABA_A_R antibody, anti-dipeptidyl-peptidase-like protein-6 (DPPX) antibody, anti-mGluR5 antibody, anti-glutamic acid decarboxylase 65 (GAD65) antibody, anti-myelin oligodendrocyte glycoprotein (MOG) antibody, anti-Ma2 antibody, anti-Dopamine 2 receptor (D_2_R) antibody, anti-Hu antibody and so on. (4) imaging and electroencephalography (EEG) data: the first results of magnetic resonance imaging (MRI) and EEG; (5) therapeutic data, including first-line immunotherapy (corticosteroids, intravenous immunoglobulin, plasma exchange) and second-line immunotherapy (cyclophosphamide, mycophenolate mofetil, and rituximab) (3, 20, 21); and (6) the scale data: the CASE and mRS scores at the time of hospital admission.

### Scale Assessment

The CASE and mRS scores were assessed simultaneously upon hospital admission. The CASE contains nine items: seizures (current status), memory dysfunction, psychiatric symptoms (delusion, hallucination, disinhibition, aggression), consciousness, language problems, dyskinesia/dystonia, gait instability and ataxia, brainstem dysfunction, and weakness. Each item was based on a 3-point grading system, except for the item ‘‘brainstem dysfunction’’, which is rated by the number of symptoms (gaze paresis, tube feeding, and ventilator care due to hypoventilation), with one point given for each symptom and a maximum of three points (16). The mRS has six grades (0-5) as follows: 0 = no symptoms, 1 = no significant disability: able to carry out all usual activities despite the presence of symptoms, 2 = Slight disability: unable to perform all usual activities but able to look after their own affairs without assistance, 3 = Moderate disability: requiring some help but able to walk without assistance, 4 = Moderately severe disability: unable to walk or attend bodily needs without assistance, 5 = Severe disability: bedridden, incontinent and requiring constant nursing care and attention (15). Two neurologists (W-HT and Z-YY), who were blinded to the research purpose, evaluated the scales independently by reviewing the detailed medical records, and consensus was achieved after discussion in any discrepant cases. If no consensus was reached, a third senior neurologist (H-SS) made the final decision.

### Statistical Analysis

Categorical variables are shown as counts and percentages and were analyzed by Pearson’s chi-squared test or Fisher’s exact test. Continuous variables are expressed as the mean ± SD or medians (quartiles), and Student’s t test or the Mann–Whitney U test was used to compare the group data accordingly. EEG and MRI data were missing in 75 (32.1%) and 8 (3.4%) patients because these examinations were either not completed or the results were not recorded in the medical records, and these patients were classified into the “unknown” group. Variables with *P* values < 0.05 in univariate analyses were subjected to multivariate logistic regression analysis with a stepwise elimination procedure to obtain an optimized model in terms of a minimal Akaike’s information criterion (AIC) value. The AE-related management decision based on the judgment of the attending clinicians was a reflection of the disease characteristics, and not all treatments were performed prior to ICU admission. Therefore, the treatment data were not included as risk factors in the multivariate logistic regression analysis. Variables with *P* values < 0.05 in the optimized multivariate regression model were used to build the final prediction model. Each model was calibrated by a calibration curve, which is actually a visualization of the Hosmer–Lemeshow test. The discriminatory ability of the models was assessed by calculating the area under the receiver operating characteristic (ROC) curves (AUC). ROC analysis was also used to calculate the optimal cutoff values, and these were determined by maximizing the Youden index. The accuracy of the optimal cutoff value was assessed by the sensitivity and specificity. Internal validation of the models was performed using bootstraps with 1000 replicates (22). To determine if any of the candidate models outperformed the others, we used the DeLong test (23) to explore each of the model pairs for a difference in the AUC values. Two-sided values of *P*<0.05 were considered statistically significant. All analyses were performed with IBM SPSS software, version 24 (SPSS Inc., Chicago, IL, USA), R software version 3.6.1 (The R Foundation for Statistical Computing, Vienna, Austria; http://www.r-project.org) and GraphPad Prism 8. We prepared this article using STROBE, which is the guideline for observational study reports.

## Results

### Clinical Characteristics of AE

**Figure 1:**
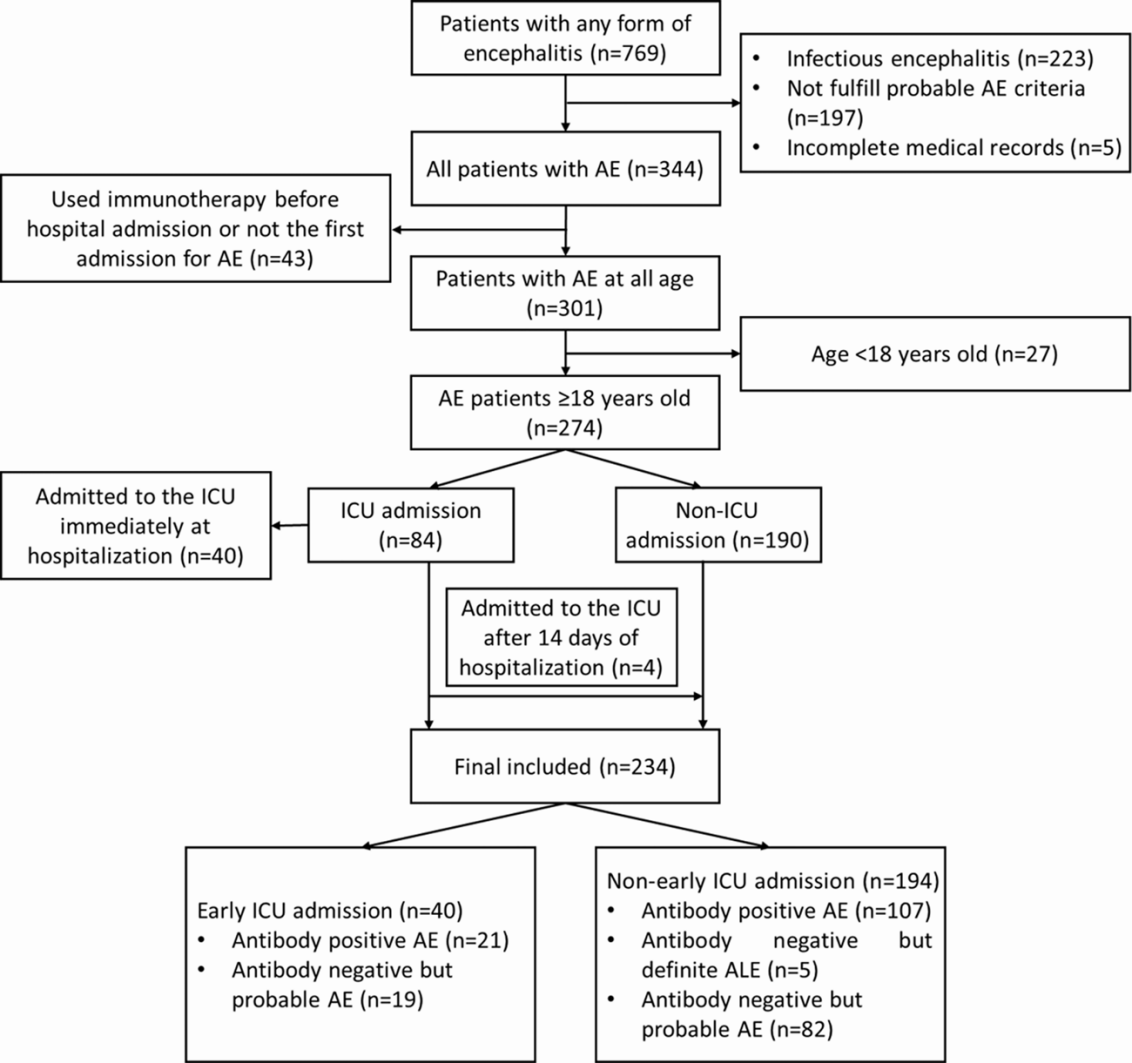
Flow chart of study patients.

From January 2013 to October 2021, a total of 769 patients with potential encephalitis were screened from the electronic database, of which 344 met the diagnosis criteria of probable or definite AE, and 234 patients were included in the final analysis after excluding the following patients: 43 patients received immunotherapy before hospitalization/non-index AE admission, 27 patients aged under 18 years old, and 40 patients admitted to the ICU immediately upon hospitalization. The flow chart of the study is shown in **Figure 1**. We did not formally calculate the sample size because the current number of patients was determined by the availability of existing data from the introduction of autoimmune encephalitis antibody testing. Of the enrolled patients, 54.7% (128/234) tested positive for neuronal antibodies, which included anti-NMDAR (n=69), LGI1 (n=14), CASPR2 (n=9), GABA_B_R (n =14), AMPA (n=3), DPPX (n=7), GAD65 (n=1), mGluR 5 (n=1), MOG (n=3), NMDAR/AMPA (n=1), LGI1/GABA_B_R (n=2), LGI1/CASPR2 (n=2), LGI1/AMPA (n=1) and GABA_B_R/MOG (n=1). Details of antibodies are shown in **Supplementary Table 3**. Five patients were autoantibody negative but were clinically diagnosed with definite autoimmune limbic encephalitis, and the remaining 101 (43.2%) patients fulfilled the criteria for autoantibody-negative but probable AE. The characteristics of the patients are summarized in **Table 1**. The median age of the 234 patients (56.0% males) was 39.0 (IQR 26.0-54.3) years. All patients were in the acute phase of the index admission, and 73.5% (172/234) had an interval of less than 1 month from symptom onset to hospital admission. The timeline of patients from hospital admission to discharge is shown in **Supplementary Figure 1**. Epilepsy was the most common initial symptom (42.3%) and was the most common symptom from onset to hospital admission (52.6%), followed by psychiatric symptoms (50.4%). Half of the patients (52.6%) had prodromal symptoms. Two hundred and one (85.9%) patients received immunotherapy, while 14.1% of patients rejected any immunotherapies due to a mild disease severity, poor economic conditions, or an intolerance of side effects. Forty patients (17.1%) deteriorated for the early of their hospitalization and were admitted to the ICU. The common direct reasons for early ICU admission were status epilepticus (32.5%, 13/40), unstable vital sign (respiratory failure or blood pressure drop) (17.5%, 7/40), severe psychiatric symptoms (15.0%, 6/40) and decreased level of consciousness (7.5%, 3/40), details are shown in **Supplementary Table 4**.

**Table 1:** Characteristics of AE patients with and without need for ICU care.

Characteristics	Total (n = 234)	Non-early ICU admission (n = 194)	Early ICU admission (n = 40)	*P*
Age	39.0 (26.0-54.3)	39.5 (25.8-56.0)	35.0 (27.0-48.0)	0.181
Gender (male)	131 (56.0%)	107 (55.2%)	24 (60.0%)	0.604
Positive antibody^a^	128 (54.7%)	107 (55.2%)	21 (52.5%)	0.862
Definite AE	133 (56.8%)	112 (57.7%)	21 (52.5%)	0.600
Comorbidities				
Tumor	17 (7.3%)	14 (7.2%)	3 (7.5%)	1.000
Hypertension	35 (15.0%)	32 (16.5%)	3 (7.5%)	0.222
Diabetes mellitus	18 (7.7%)	16 (8.2%)	2 (5.0%)	0.707
Autoimmune disease	12 (5.1%)	9 (4.6%)	3 (7.5%)	0.724
Interval from symptoms onset to hospital admission (months)				0.024
>3	12 (5.1%)	12 (6.2%)	0 (0.0%)	
1-3	50 (21.4%)	46 (23.7%)	4 (10.0%)	
≤1	172 (73.5%)	136 (70.1%)	36 (90.0%)	
Prodromal symptoms	123 (52.6%)	93 (47.9%)	30 (75.0%)	0.003
Onset symptoms				
Epilepsy	99 (42.3%)	79 (40.7%)	20 (50.0%)	0.296
Psychiatric/cognition disturbances	102 (43.6%)	85 (43.8%)	17 (42.5%)	1.000
Consciousness disorders	14 (6.0%)	11 (5.7%)	3 (7.5%)	0.938
Symptoms from onset to hospital admission				
Epilepsy	123 (52.6%)	98 (50.5%)	25 (62.5%)	0.223
Short-term memory dysfunction	65 (27.8%)	58 (29.9%)	7 (17.5%)	0.124
Psychiatric symptoms	118 (50.4%)	90 (46.4%)	28 (70.0%)	0.009
Consciousness disorders	52 (22.2%)	33 (17.0%)	19 (47.5%)	< 0.001
Language dysfunction	38 (16.2%)	30 (15.5%)	8 (20.0%)	0.483
Extrapyramidal symptoms	13 (5.6%)	10 (5.2%)	3 (7.5%)	0.833
Autonomic dysfunction	12 (5.1%)	9 (4.6%)	3 (7.5%)	0.724
Sleep disorders	26 (11.1%)	23 (11.9%)	3 (7.5%)	0.602
CSF test				
Elevated CSF pressure^b^	59 (25.2%)	40 (20.6%)	19 (47.5%)	0.001
Elevated CSF WBC count^b^	116 (49.6%)	85 (43.8%)	31 (77.5%)	<0.001
Elevated CSF total protein^b^	81 (34.6%)	66 (34.0%)	15 (37.5%)	0.716
Blood test				
Elevated WBC count^b^	61 (26.1%)	46 (23.7%)	15 (37.5%)	0.078
Anemia^b^	77 (32.9%)	65 (33.5%)	12 (30.0%)	0.716
Platelet (×10^9/L)	226.0 (188.0-272.0)	224.0 (188.8-272.0)	235.5 (185.5-277.8)	0.801
Elevated fasting blood glucose^b^	49 (20.9%)	31 (16.0%)	18 (45.0%)	< 0.001
Impaired hepatic function	42 (17.9%)	33 (17.0%)	9 (22.5%)	0.497
Hypokalemia^b^	26 (11.1%)	21 (10.8%)	5 (12.5%)	0.783
Na^+^				0.006
Normal ^b^	195 (83.3%)	168 (86.6%)	27 (67.5%)	
Decreased	33 (14.1%)	23 (11.9%)	10 (25.0%)	
Increased	6 (2.6%)	3 (1.5%)	3 (7.5%)	
Cl^-^				0.060
Normal ^b^	175 (74.8%)	150 (77.3%)	25 (62.5%)	
Decreased	55 (23.5%)	42 (21.6%)	13 (32.5%)	
Increased	4 (1.7%)	2 (1.0%)	2 (5.0%)	
Hypocalcemia^b^	36 (15.4%)	26 (13.4%)	10 (25.0%)	0.089
Creatinine (umol/L)	65.5 (55.0-76.0)	66.5 (55.0-76.0)	64.0 (53.3-77.0)	0.655
Uric acid (umol/L)	258.7 (191.5-321.5)	264.0 (202.5-324.3)	231.7 (150.8-304.6)	0.064
EEG				0.031
Normal	68 (29.1%)	63 (32.5%)	5 (12.5%)	
Abnormal^c^	91 (38.9%)	70 (36.1%)	21 (52.5%)	
Unknown	75 (32.1%)	61 (31.4%)	14 (35.0%)	
MRI				0.052
Normal	90 (38.5%)	76 (39.2%)	14 (35.0%)	
Abnormal^c^	136 (58.1%)	114 (58.8%)	22 (55.0%)	
Unknown	8 (3.4%)	4 (2.1%)	4 (10.0%)	
Treatment				
Immunotherapy	201 (85.9%)	163 (84.0%)	38 (95.0%)	0.082
First-line	200 (85.5%)	162 (83.5%)	38 (95.0%)	0.082
Second-line	7 (3.0%)	4 (2.1%)	3 (7.5%)	0.184
ASMs	124 (53.0%)	95 (49.0%)	29 (72.5%)	0.009
Scale				
mRS	2 (1-3)	2 (1-3)	3 (2-4)	< 0.001
CASE	4 (2-5)	3 (2-5)	7 (5-11)	< 0.001

a Antibodies against cell-surface, synaptic, or onconeural protein.

b Normal values: CSF pressure (80-180 mmH2O), CSF WBC count (≤5/mm3), CSF protein (150-450 mg/L), blood WBC ((4-10) ×10^9/L ), Na+ (135-145 mmol/L) and Cl- (98-110 mmol/L). anemia was defined as < 120 g/L in females and children and < 135 g/L in males; elevated fasting blood glucose was defined as >6.1mmol/L; hypokalemia was defined as < 3.5 mmol/L; hypocalcemia was defined as < 2.15 mmol/L.

c Abnormal EEG results: epileptic discharge, delta brush, or slow wave. Abnormal brain MRI results: brain MRI hyperintense signal on T2-weighted fluid-attenuated inversion recovery sequences highly restricted to one or both medial temporal lobes or in multifocal areas involving gray matter, white matter, or both compatible with demyelination or inflammation.

### Factors Associated With Early ICU Admission Among AE Patients

**Figure 2:**
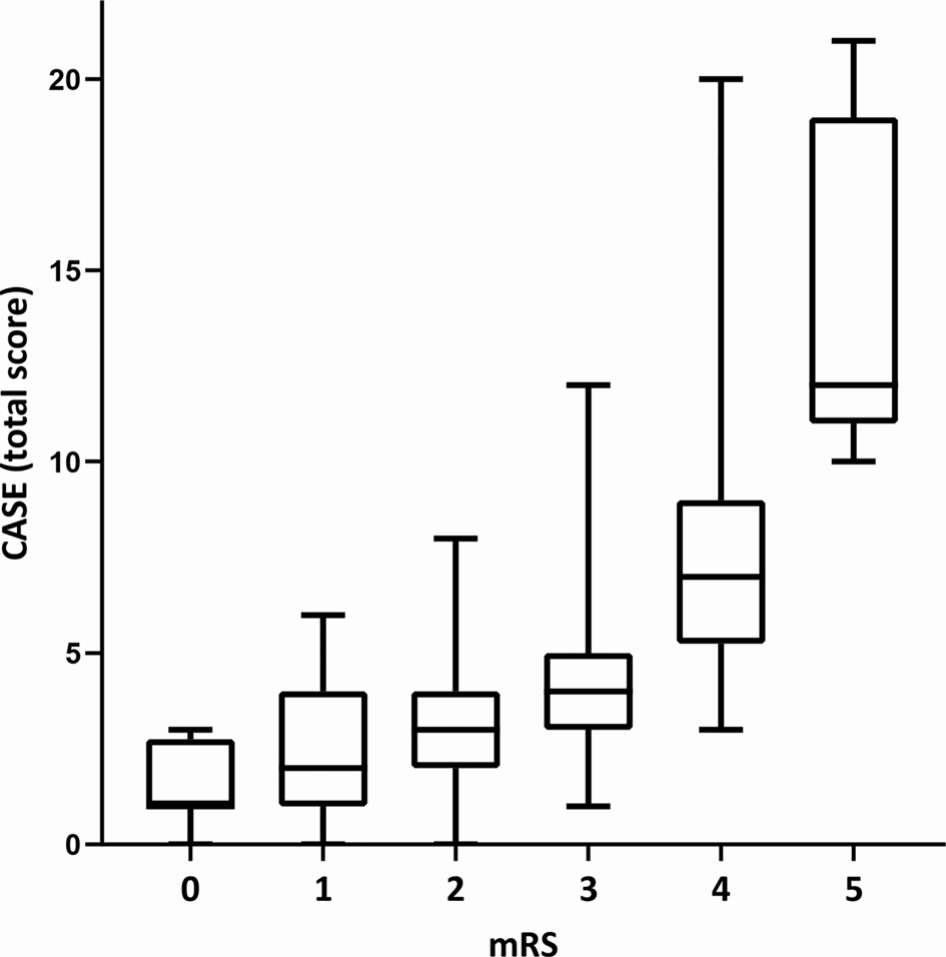
The total CASE score according to the mRS at the time of hospital admission. CASE, the Clinical Assessment Scale for Autoimmune Encephalitis. mRS, the modified Rankin scale. The CASE and mRS scores were statistically correlated (r = 0.642, P < 0.001).

As shown in **Table 1**, the total CASE and mRS scores were both significantly associated with ICU admission (*P*<0.001). We found that the CASE and mRS scores were statistically correlated (r=0.642, *P*<0.001) (**Figure 2**), which was consistent with previous studies (7, 17, 18). Other variables that were significant associated with admission to the ICU included time from symptom onset to hospital admission of less than 1 month (*P*=0.024), prodromal symptoms (*P*=0.003); symptoms from onset to hospital admission: psychiatric symptoms (*P*=0.009), consciousness disorders (*P*<0.001); laboratory tests: elevated CSF pressure (*P*=0.001), elevated CSF WBC count (*P*<0.001), elevated fasting blood glucose (*P*<0.001), Na^+^ (*P*=0.006); abnormal or unknown EEG results (*P*=0.031), and anti-seizure medications (ASMs) therapy (*P*=0.009).

### Risk Models for Prediction Early ICU Admission

#### Model Construction

**Figure 3:**
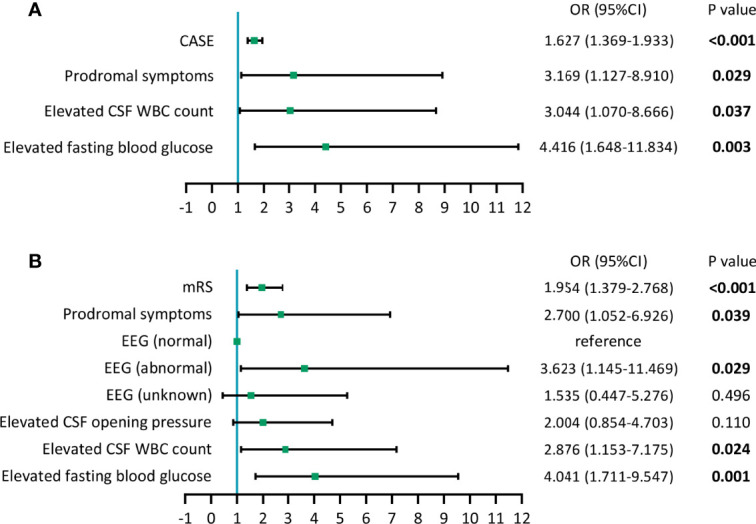
Forest plots of models of multivariate logistic regression analysis. **(A)** Results of multivariate logistic regression analysis containing CASE. **(B)** Results of multivariate logistic regression analysis containing mRS.

Next, we conducted two multivariate logistic regression analyses. One included the CASE score and the variables with *P*<0.05 in the univariate analysis, and the other included the mRS score and variables with *P*<0.05 in the univariate analysis. The results of the optimized multivariate regression model after the variable selection are shown as forest plots (**Figure 3**). As seen from the forest plots, there were four significant independent predictors (CASE, prodromal symptoms, elevated fasting blood glucose and elevated CSF WBC count) in the CASE model and five (mRS, prodromal symptoms, abnormal EEG results, elevated fasting blood glucose and elevated CSF WBC count) in the mRS model.

Based on the results of the multivariate analysis, we developed the following four candidate predictive models:

Model 1: CASE: CASE alone;

Model 2: CASE-plus: CASE + prodromal symptoms + elevated fasting blood glucose + elevated CSF WBC count;

Model 3: mRS: mRS alone;

Model 4: mRS-plus: mRS + prodromal symptoms + abnormal EEG results + elevated fasting blood glucose + elevated CSF WBC count

#### Model Evaluation

##### Discrimination

**Figure 4:**
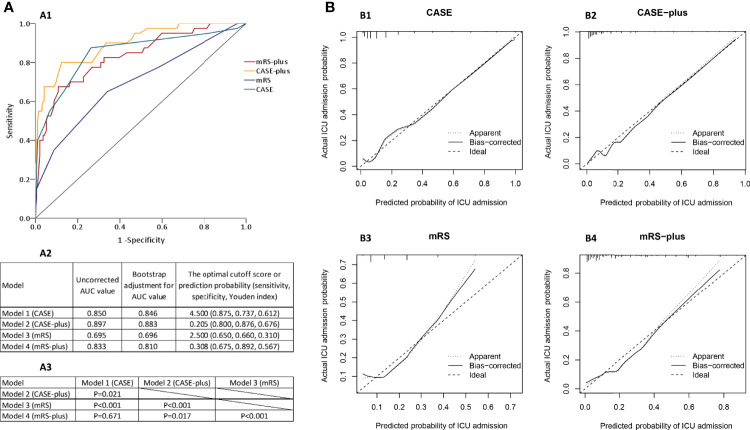
Risk prediction models for early ICU admission in patients with AE. **(A1)** Receiver operating characteristic (ROC) curves of the four models for predicting early ICU admission. **(A2)** Uncorrected and the bootstrap-adjusted AUC values and optimal cutoff values of the four models. The AUC values of CASE, CASE-plus, mRS, mRS-plus were 0.850 (95% CI 0.773-0.927, P<0.001), 0.897 (95% CI 0.842-0.953, P<0.001), 0.695 (95% CI 0.599-0.792, P<0.001), and 0.833 (95% CI 0.760-0.906, P<0.001), respectively. **(A3)** P values for the pairwise comparison of original AUC values of the four models. **(B)** The calibration curves of the four models in predicting ICU admission. The y-axis represents the actual probability of ICU admission, and the x-axis represents the predicted probability of ICU admission. A perfect model would fully match the 45° ideal line.

The ROC curves for each model are presented in **Figure 4A1**. **Figure 4A2** shows the uncorrected AUC values and the bootstrapped optimism corrected AUC values for each model. The bootstrap-adjusted AUC values were similar to the uncorrected AUC values. The optimal cutoff scores, which were derived from the ROC analysis, of Model 1 and Model 3 were 4.5 and 2.5, respectively. We also summarized the sensitivity and specificity in estimating the risk of ICU admission using 4.5 and 2.5 as the cutoff values (**Figure 4A2**). To determine if any of the four candidate models outperformed the others, we used DeLong’s test (23) to test each of the four correlated possible model pairs for a difference in predicting the uncorrected AUC scores. We found a significant difference in the discriminant ability of each pair (*P*<0.05), except for Model 1 and Model 4 (*P*=0.671), and the *P* values are listed in **Figure 4A3**. By combining **Figure 4A**, we can conclude that Model 2 performed best, followed by Model 1, Model 4 and Model 3, with AUCs of 0.897 (95% CI 0.842-0.953, P<0.001), 0.850 (95% CI 0.773-0.927, P<0.001), 0.833 (95% CI 0.760-0.906, P<0.001) and 0.695 (95% CI 0.599-0.792, P<0.001), respectively (**Figure 4A2**).

##### Calibration

The Hosmer–Lemeshow test of these four models showed (with *P* values of 0.14, 0.44, 0.35 and 0.40 for Models 1, 2, 3 and 4, respectively) that the calibrations of the four models were adequate and that the models were correctly specified. The apparent and bootstrapped calibration curves for each model (**Figure 4B**) showed the excellent agreement between the observed outcomes and the predictions, with predicted probabilities positioned on or around a 45° line of the plot.

## Discussion

In this retrospective study of critically ill and noncritically ill patients with AE who were treated in a tertiary hospital, we investigated the association between the patient characteristics and early ICU admission. In the univariate analysis, both the CASE and mRS scores were significantly associated with ICU admission. Next, we developed four risk prediction models: CASE, CASE-plus (CASE plus = CASE + prodromal symptoms + elevated fasting blood glucose + elevated CSF WBC count), mRS, and mRS-plus (mRS plus = mRS + prodromal symptoms + abnormal EEG results + elevated fasting blood glucose + elevated CSF WBC count). Among the four models, the CASE-plus model demonstrated the best performance.

CASE is the first clinical severity scale that was specifically designed for the various syndromes of AE. Although lacking large-scale validation, Cai and Zhang et al. proved that CASE performed well and had a significant positive correlation with mRS in two groups of Chinese patients with antibody-positive AE (17, 18). As a comprehensive scale covering multiple domains of AE, CASE has inherent advantages when compared to mRS. First, the detailed assessment of various specific clinical manifestations allows CASE to represent the overall severity of the disease, especially in patients with nonmotor symptoms and who develop common intensive care signs, such as status epilepticus, coma and mechanical ventilation due to central hypoventilation, and these variables are not included in mRS. Second, the total score of CASE ranges from 0 to 27, and mRS is a 6-point scale (15, 16). This discrepancy makes CASE more precise and sensitive in differentiating the severity of disease within the same range of measurements that are also defined by mRS (16). In our study, total CASE scores of 4.5/27 and total mRS scores of 2.5/6 at the time of hospitalization were the optimal cutoff values in predicting ICU admission; to some extent, these values reflect the early predictive value and sensitivity of the CASE score compared with the mRS score. The cutoff value of the total CASE score also implied that patients with multiple moderate to severe symptoms at the time of admission were more likely to progress to critical conditions. In such cases, quantified symptoms can serve as an alert, which allows patients to receive advanced treatment in a timely manner. Several limitations of CASE may also exist. First, CASE is more complicated and time-consuming than mRS, and it is difficult to use CASE to evaluate some symptoms in specific situations. For example, in sedated patients, the assessment of symptoms such as language and memory can be challenging. Second, the score of each item is unweighted, and CASE is a three-point scale, which may be unfair in assessing some fatal symptoms, such as central hypoventilation. Overall, the total CASE score might be a better optimal predictor of early ICU admission than the mRS score because of its more comprehensive characteristics.

CASE and mRS reflect the pro tempore status of the patient; however, other symptoms can develop in the early course of AE. Also, laboratory and imaging abnormalities may contribute to disease deterioration. We then included more variables associated with AE to screen out other potential risk factors for ICU admission. We found that prodromal symptoms, abnormal EEG results, elevated fasting blood glucose and elevated CSF WBC count were independent predictors in the multivariable analysis. In our study, 52.6% of patients had prodromal symptoms, which is consistent with previous studies (34%-62.8%) (2, 24, 25). Prodromal symptoms are nonspecific, vary in presentation and, more importantly, indicate infection (26). In fact, infections have long been suspected to play a role in triggering or enhancing the autoimmune process (26, 27). Accumulating evidence suggests that viral infections may be associated with the development of AE (28, 29). In other autoimmune diseases, such as myasthenia gravis (MG) (30) and Guillain–Barre syndrome (GBS) (31, 32), patients who triggered by infection often have a higher ICU admission rate and a more unfavorable prognosis. Elevated CSF WBC count, an indicator of inflammation within the central nervous system (33), was also found to be associated with ICU admission in another study (6). Previously elevated fasting blood glucose might have been ignored as a variable in AE. However, in the setting of acute inflammation, stress hyperglycemia is often observed, which is common in critically ill patients and appears to be a marker of disease severity (34). We found that patients with elevated fasting glucose levels were four times more likely to be admitted to the ICU than patients without elevated fasting glucose levels. However, only 11 of the 49 patients with abnormal fasting glucose levels were diagnosed with diabetes or with an impaired fasting glucose tolerance, indicating that the majority of these patients had acute glucose instability. Therefore, it is feasible for an abnormal fasting blood glucose level at admission to serve as an indicator for the early identification of critical illness in AE cases. Thus, we postulate that infection-triggered and multisystem-involved patients with AE may suffer a more severe disease index.

To generate prediction models with a higher sensitivity, we integrated the above risk factors into CASE and mRS. We found that the predictability of each ICU admission model (except for mRS), as measured by the area under the ROC curve, was more than 0.80. Models containing the CASE score performed better than those containing the mRS score. The CASE-plus and mRS-plus model performed better than the CASE or mRS models, respectively. The best-performing model was the CASE-plus model. This result confirmed that considering both clinical phenotypes and biological disturbances would precisely predict disease progression.

Several limitations should be noted in our study. First, the retrospective nature of the design makes it difficult to control for confounding factors and may lead to possible information bias. AE is a disease that has gradually received attention with the development of antibody detection technology in recent years. Many of the large studies on AE are also retrospective (35, 36), and the results are repeatable. The assessment of CASE was performed retrospectively, and there would inevitably be a small number of patients with incomplete documentation of some items, such as the grading of dyskinesia/dystonia and memory dysfunction. For these patients, we carefully reviewed the medical records, and if no relevant symptoms were recorded throughout the course of the disease, it was considered not present. Nonetheless, CASE has been used in retrospective studies (17, 18, 37) and it is feasible to consider the results of our study are reliable. Second, this is a single-center study, and selection bias may exist. For example, the ICU admission rate was lower than that in previous studies in Western countries (13, 38). As a national tertiary hospital, Tongji Hospital receives a wide coverage and a great number of patients, which makes the patients representative of the general population. In fact, the overall severity and severity distribution of the patients in our study are comparable to those from several domestic studies (17, 18, 39, 40). Third, although the internal validation in our study showed good efficacy, this study was not externally validated. Generalizing the conclusions of this study requires validation in further external datasets, and a prospective, multicenter study with a larger sample size will be necessary in the future.

To the best of our knowledge, this is the first study to integrate the scale-based disease severities of patients with AE into predictive models for ICU admission. Both the CASE and mRS models could accurately predict the risk of ICU admission in AE patients, but the CASE model performed better. Patients with CASE scores ≥5 were more likely to be admitted to the ICU. Adding prodromal symptoms, elevated fasting blood glucose and CSF WBC count to the CASE model could improve the predictive ability of the existing grading scale.

## Data Availability Statement

The data supporting the findings of this study are available from the corresponding authors, upon reasonable request.

## Ethics Statement

The studies involving human participants were reviewed and approved by the Institutional Ethics Board of Tongji Hospital Tongji Medical College of Huazhong University of Science and Technology (ID: TJ-IRB20211221). Written informed consent from the participants’ legal guardian/next of kin was not required to participate in this study in accordance with the national legislation and the institutional requirements.

## Author Contributions

CW designed the study, acquired data, performed the analysis and drafted the work; YF analyzed and interpreted of the data; YZ and HW acquired data; SH designed the study. SH and SZ led the study, critically reviewed and revised the manuscript. All authors contributed to the article and approved the submitted version.

## Funding

This study was supported by the Hubei Technological Innovation Special Fund (CN) [Grant NO.2019ACA132] and Hubei Natural Science Foundation Grant NO. 2020CFB805.

## Conflict of Interest

The authors declare that the research was conducted in the absence of any commercial or financial relationships that could be construed as a potential conflict of interest.

## Publisher’s Note

All claims expressed in this article are solely those of the authors and do not necessarily represent those of their affiliated organizations, or those of the publisher, the editors and the reviewers. Any product that may be evaluated in this article, or claim that may be made by its manufacturer, is not guaranteed or endorsed by the publisher.

## Abbreviations

ADEM, acute disseminated encephalomyelitis; AE, autoimmune encephalitis; AIC, Akaike’s information criterion; ALE, autoimmune limbic encephalitis; AMPA, α-amino-3-hydroxy-5-methyl-4-isoxazolepropionic acid; ASMs, anti-seizure medications; AUC, area under the receiver operating characteristic (ROC) curve; CASE, the clinical assessment scale in autoimmune encephalitis; CASPR, contactin associated protein; CSF, cerebrospinal fluid; D_2_R, Dopamine 2 receptor; DPPX, dipeptidyl-peptidase-like protein; EEG, electroencephalography; GABA, gamma-aminobutyric acid; GAD, glutamic acid decarboxylase; GBS, Guillain–Barre syndrome; ICU, intensive care unit; LGI1, leucine-rich glioma inactivated 1; MG, myasthenia gravis; mGluR, metabotropic glutamate receptor; MOG, myelin oligodendrocyte glycoprotein; MRI, magnetic resonance imaging; mRS, the modified Rankin Scale; NMDA, N-methyl-D-aspartate; WBC, white blood cell.
