# Supplementary material for: Risk Prediction Models for Early ICU Admission in Patients With Autoimmune Encephalitis: Integrating Scale-Based Assessments of the Disease Severity
Source: Front Immunol. 2022 Jun 10;13:916111. doi: 10.3389/fimmu.2022.916111 (PMC9226454; doi:10.3389/fimmu.2022.916111)
Supplement: Supplementary file 1 [file DataSheet_1.docx]

**Supplementary Table 1. Diagnostic criteria of the patients**

| AE syndrome | Diagnostic criteria |
| --- | --- |
| Definite AE | 1. Fulfillment of the criteria for possible AE^*^  2. Positive for antibodies against cell-surface, synaptic, or onconeural proteins |
| Definite ALE (all of the four criteria) | 1. Subacute onset (rapid progression of less than 3 months) of working memory deficits, seizures, or psychiatric symptoms suggesting involvement of the limbic system  2. Bilateral brain abnormalities on T2-weighted fluid-attenuated inversion recovery MRI highly restricted to the medial temporal lobes  3. At least one of the following:  1) CSF pleocytosis (white blood cell count of more than five cells per mm^3^)  2) EEG with epileptic or slow-wave activity involving the temporal lobes  4. Reasonable exclusion of alternative causes |
| Autoantibody-negative but probable AE (all of the four criteria) | 1. Rapid progression (less than 3 months) of working memory deficits (short-term memory loss), altered mental status, or psychiatric symptoms  2. Exclusion of well-defined syndromes of autoimmune encephalitis (e.g, typical limbic encephalitis, Bickerstaff ’s  brainstem encephalitis, acute disseminated encephalomyelitis)  3. Absence of well-characterized autoantibodies in serum  and CSF, and at least two of the following criteria:  1) MRI abnormalities suggestive of AE  2) CSF pleocytosis, CSF-specific oligoclonal bands or elevated CSF IgG index or both  3) Brain biopsy showing inflammatory infiltrates and excluding other disorders (e.g, tumor)  4. Reasonable exclusion of alternative causes |

^*^ Diagnostic criteria of possible autoimmune encephalitis: 1. Subacute onset (rapid progression within less than 3 months) of working memory deficits (short-term memory loss), altered mental status, or psychiatric symptoms. 2. At least one of the following: 1) new focal CNS findings, 2) seizures not explained by a previously known seizure disorder, 3) CSF pleocytosis (a white blood cell count of more than five cells per mm³), or 4) MRI features suggestive of encephalitis. 3. Reasonable exclusion of alternative causes. Abbreviations: AE=autoimmune encephalitis, ALE=autoimmune limbic encephalitis, CSF=cerebrospinal fluid, MRI=magnetic resonance image, IgG=immunoglobulin G

Supplementary Table 2.Ancillary investigations of other causes

| Other causes | Ancillary investigations |
| --- | --- |
| Infectious | Viruses (HSV, Enteroviruses, Paramyxoviruses…), Bacteria (Small bacteria, Spirochetes, Other bacteria), Tuberculosis, Parasites, Fungi/NGS |
| Metabolic | Renal, liver, bone & thyroid profiles |
|  | Arterial blood gas analysis |
|  | Plasma and CSF lactate, ammonia, pyruvate, amino acids, very long-chain fatty acids, urinary organic acids |
|  | Porphyrins: blood/urine/faeces |
|  | Biopsy: skin, lymph node, peripheral nerve/muscle |
| Vascular | CT or MRI head with venogram and/or angiogram |
| Neoplastic | MRI brain and MR spectroscopy |
|  | CSF cytological analysis |
|  | Brain and meningeal biopsy |
|  | CT chest/abdomen/pelvis |
|  | LDH, IgG/A/M, protein electrophoresis, urinary Bence-Jones protein (in adults), bone marrow trephine |
| Toxic | Blood film; blood or urine levels of alcohol, paracetamol, salicylate, tricyclic, heavy metals |
|  | Urinary illicit drug screen |
| Septic Encephalopathy | Serum microbiological cultures, serology and PCR |
| Creutzfeldt-Jakob disease | EEG, MRI, CSF(14-3-3 protein), serum (S100 protein), brain biopsy |
| Mitochondrial diseases | Lactic acid, pyruvate minimum exercise test, CT or MRI head, Mitochondrial DNA Analysis |
| Inflammatory encephalitis | FBC, ESR, CRP, ANA, ENA, dsDNA, ANCA, C3, C4, lupus anticoagulant, cardiolipin, thyroglobulin, thyroperoxidase antibodies, ferritin, fibrinogen, trigylcerides |
|  | Serum and CSF ACE, Serum 25OH Vitamin D, 24hr urinary calcium |
|  | Whole body CT |
|  | Biopsy: Brain, meninges, skin, lymph node, peripheral nerve/muscle |

Abbreviations: HSV herpes simplex virus; NGS Next Generation Sequencing; MRI magnetic resonance imaging; ASO antistreptolysin; PCR polymerase chain reaction; CSF cerebrospinal fluid; FBC full blood count; ESR erythrocyte sedimentation rate; CRP C-reactive protein; ANA antinuclear antibodies; ENA extraneuclear antibodies; dsDNA double stranded deoxyribonucleic acid antibodies; C3/4 complement; ACE angiotensin converting enzyme; CT computed tomography; LDH lactate dehydrogenase; IgG/M/A immunoglobulin; PET positron emission tomography; CNS central nervous system.

Supplementary Table 3. Details of antibodies in antibody-positive patients.

Supplementary Table 3.1 Results of patients with single antibody in this study.

| Antibody type | Total Patients (121) | Serum positive only  (31) | CSF positive only  (29) | Both serum and CSF positive  (61) | Serum (92) | | | CSF (90) | | |
| --- | --- | --- | --- | --- | --- | --- | --- | --- | --- | --- |
|  |  |  |  |  | + (30) | ++ (43) | +++ (19) | + (21) | ++ (43) | +++ (26) |
| NMDA | 69 (57%) | 13 (42%) | 21 (72%) | 35 (57%) | 17 (57%) | 21 (49%) | 10 (53%) | 12 (57%) | 27 (63%) | 17 (65%) |
| LGI1 | 14 (12%) | 2 (6%) | 3 (10%) | 9 (15%) | 1 (3%) | 7 (16%) | 3 (16%) | 3 (14%) | 8 (19%) | 1 (4%) |
| GABAB | 14 (12%) | 4 (13%) | 2 (7%) | 8 (13%) | 5 (17%) | 3 (7%) | 4 (21%) | 0 (0%) | 3 (7%) | 7 (27%) |
| CASPR2 | 9 (7%) | 8 (26%) | 0 (0%) | 1 (2%) | 3 (10%) | 6 (14%) | 0 (0%) | 1 (5%) | 0 (0%) | 0 (0%) |
| DPPX | 7 (6%) | 3 (10%) | 1 (3%) | 6 (10%) | 0 (0%) | 4 (9%) | 2 (11%) | 2 (10%) | 2 (5%) | 0 (0%) |
| AMPA | 3 (2%) | 1 (3%) | 0 (0%) | 2 (3%) | 2 (7%) | 1 (2%) | 0 (0%) | 1 (5%) | 0 (0%) | 1 (4%) |
| MOG | 3 (2%) | 0 (0%) | 1 (3%) | 2 (3%) | 2 (7%) | 0 (0%) | 0 (0%) | 2 (10%) | 1 (2%) | 0 (0%) |
| mGluR5 | 1 (1%) | 0 (0%) | 1 (3%) | 0 (0%) | 0 (0%) | 0 (0%) | 0 (0%) | 0 (0%) | 1 (2%) | 0 (0%) |
| GAD65 | 1 (1%) | 0 (0%) | 0 (0%) | 1 (2%) | 0 (0%) | 1 (2%) | 0 (0%) | 0 (0%) | 1 (2%) | 0 (0%) |

Supplementary Table 3.2. Results of patients with multiple antibodies in this study.

| Multiple antibodies | Total patients (7) | Titer of the first patient | Titer of the second patient |
| --- | --- | --- | --- |
| LGI1/CASPR2 | 2 | CSF ++/+++;  serum -/- | CSF ++/-; serum +/+ |
| LGI1/GABAB | 2 | CSF ++/++  serum -/- | CSF -/-;  serum ++/+ |
| NMDA/AMPA | 1 | CSF ++/++;  Serum +++/++ | - |
| LGI1/AMPA | 1 | CSF -/++; serum +/++ | - |
| GABAB/MOG | 1 | CSF ++/-; serum -/++ | - |

Supplementary Table 4. The direct reasons for early ICU admission.

| Reasons | Patients (40) |  |
| --- | --- | --- |
| Status epilepticus | 13 (32.50%) |  |
| Unstable vital sign (respiratory failure or blood pressure drop) | 7 (17.50%) |  |
| Severe psychiatric symptoms | 6 (15.00%) |  |
| Decreased level of consciousness | 3 (7.50%) |  |
| Decreased level of consciousness and intractable epilepsy | 3 (7.50%) |  |
| Decreased level of consciousness and unstable vital sign | 4 (10.00%) |  |
| Decreased level of consciousness and severe psychiatric symptoms | 1 (2.50%) |  |
| Status epilepticus and unstable vital sign | 1 (2.50%) |  |
| Status epilepticus and severe psychiatric symptoms | 1 (2.50%) |  |
| Decreased level of consciousness and intractable epilepsy or status Epilepticus and unstable vital sign | 1 (2.50%) |  |

Supplementary Figure 1. Timeline of patients from hospital admission to discharge.


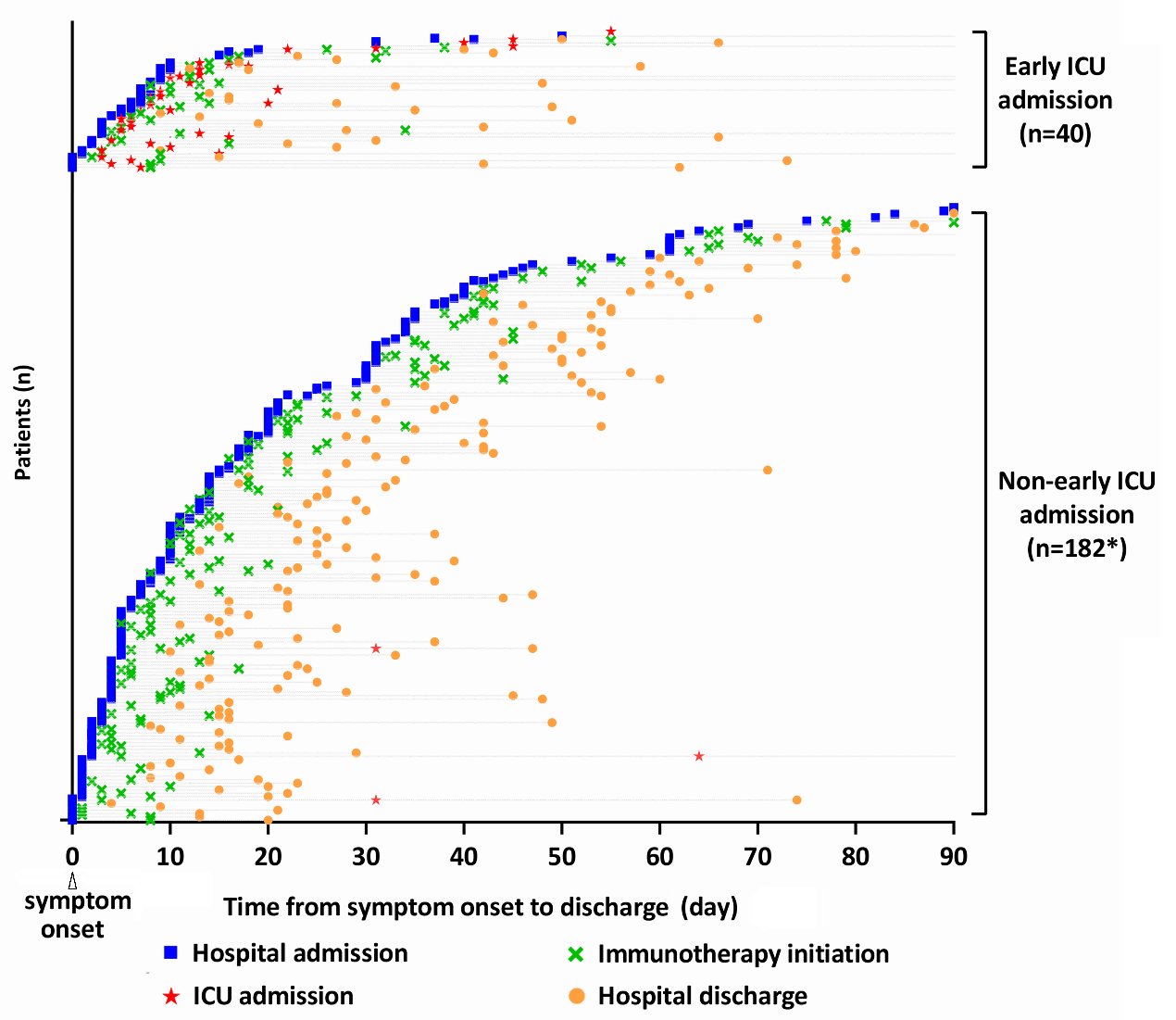


Note: * Twelve patients had more than 90 days from symptom onset to hospital admission and were therefore not shown in the figure.
